# Chemical Composition, Antioxidant, Anticholinesterase and Anti-Tyrosinase Activities of Essential Oils of Two* Sideritis *Species from Turkey

**DOI:** 10.22037/ijpr.2019.1100657

**Published:** 2019

**Authors:** Ebru Deveci, Gülsen Tel-Çayan, Özlem Usluer, Mehmet Emin Duru

**Affiliations:** a *Department of Chemistry, Faculty of Sciences, Muğla Sıtkı Koçman University, 48000 Muğla, Turkey.*; b *Department of Chemistry and Chemical Processing Technologies, Muğla Vocational School, Muğla Sıtkı Koçman University, 48000 Muğla, Turkey.*; c *Department of Chemistry, University of Massachusetts, Amherst, 01003 Massachusetts, United States.*

**Keywords:** Sideritis species, Essential oil, Medicinal plants, Antioxidant activity, Anticholinesterase activity, Anti-tyrosinase activity

## Abstract

*Sideritis* species have been known as medicinal plants since ancient times, and used as tea in Mediterranean countries such as Turkey, Greece, and Spain. They are also used for the treatment of several ailments such as a cough, common cold, and gastrointestinal disorders. The aim of the present study was to perform the chemical composition, antioxidant, anticholinesterase, and anti-tyrosinase activities of the essential oils of *Sideritis albiflora* and *S. leptoclada*. *β*-caryophyllene (21.2%) and Germacrene D (17.9%) were identified as the major compounds in *S. albiflora* and *S. leptoclada* essential oils, respectively. The essential oil of *S. albiflora* showed the highest lipid peroxidation inhibitory (IC_50_: 73.8 ± 0.8 µg/mL), DPPH free radical scavenging (28.3±0.1%), ABTS cation radical scavenging (IC_50_: 50.6 ± 1.0 µg/mL), reducing power (A_0.05_: 181.7 ± 0.6 µg/mL), acetylcholinesterase (22.1 ± 0.4%), butyrylcholinesterase (IC_50_: 157.2 ± 0.9 µg/mL) and tyrosinase (15.2 ± 0.4%) inhibitory activities. Moreover, *S. albiflora *essential oil had rich total phenolic and flavonoid contents indicating 41.5 ± 0.8 µg PEs/mg and 21.4 ± 1.0 µg QEs/mg respectively. This study suggests that consumption of *Sideritis* species as tea may protect one against melanogenesis, amnesia, and oxidative stress without any observable side effect.

## Introduction

Since ancient times, essential oils extracted from various plants have performed an important role in many different areas such as pharmaceutical, food, and cosmetic industries (1). Literature surveys have shown that essential oils exhibit antioxidant, antibacterial, anticancer, antifungal, antiviral, antimycotic, antiparasitic, anticholinesterase, anti-inflammatory, cytotoxic, and antimutagenic activities (2-7).

*Sideritis* genus is a member of Lamiaceae family and comprises at least 150 species of annual or perennial herbs and shrubs. Many species of *Sideritis* genus are found in the Mediterranean area (8). *Sideritis* species are represented by 46 species and 53 taxa in Turkey out of which 39 taxa being one of the most endemic with a 78.2 % endemism rate (9,10). *Sideritis* species are known as “mountain tea” and consumed as traditional teas and herbal medicines in Mediterranean countries (11, 12).

*Sideritis* species are effective for the treatment of gastrointestinal disturbances, cold, cough, and flu symptoms. Also, these species showed a wide spectrum of biological properties such as antimicrobial, anti-inflammatory, antiulcer, analgesic, antioxidant, insecticidal, antirheumatic and cytotoxic effects (13-19).

Due to the importance of *Sideritis *species in phytotheraphy, it is necessary to investigate their chemical compositions and biological activities in detail. Up to now, the essential oil compositions of several *Sideritis* species have been studied (20-25). According to the literature data, antimicrobial activity and the phenolic composition of the methanol extract of *S. leptoclada* were studied (21, 26, 27). Also, antioxidant activity of the water extract of *S. leptoclada* was reported by Ayar-Kayali *et al.* (28). In addition, the chemical composition of the essential oil of *S. albiflora* was determined by Topçu *et al.* (21). 

Free radicals, causing oxidative stress, damage the cell membranes, lipids, proteins, nucleic acids and DNA, and results in many diseases such as cancer, coronary diseases, cataracts, liver damage and diabetes. Antioxidants are substances delay or inhibit the oxidative stress (29). For this reason, antioxidants play an important role in the prevention of diseases caused by free radical protection and human health. Some synthetic antioxidants such as BHA (butylated hydroxyanisole), BHT (butylated hydroxytoluene), PG (propyl gallate) and TBHQ (tert-butylhydroquinone) are used in the food and pharmaceutical industries but they have been found to have toxic effects (30). For this reason, there is a tendency to obtain compounds having antioxidant potential from natural products.

In many studies, it has been found that oxidative stress is associated with the pathogenesis of neurodegenerative disorder Alzheimer’s disease (AD) and high consumption of antioxidants in daily life reduces the risk of AD. Furthermore, the inhibition of the acetylcholinesterase (AChE) and butyrylcholinesterase (BChE) enzymes is another method used in the treatment of this disease (31). Galantamine, tacrine and rivastigmine which are used as cholinesterase inhibitors for treatment of AD have side effects such as liver damage bradycardia, gastrointestinal disturbance and hepatotoxicity (32,33). Therefore, it is necessary to search for new natural products which exhibit efficient enzyme inhibition but have no side effects.

To date, no reports about antioxidant, anticholinesterase and anti-tyrosinase activities of the essential oils of *S. albiflora* and *S. leptoclada* have been found. The aim of this study was to quantify the chemical composition, antioxidant and enzyme inhibitory activities of essential oils of *S. albiflora* and *S. leptoclada* as well as their phenolic and flavonoid contents. 

## Experimental

*Plant materials*

The aerial parts of *S. albiflora* Hub.-Mor. and *S. leptoclada *O. Schwarz & P.H. Davis were collected from Muğla, Turkey in July 2013. The plants were identified by Dr. Hasan Yıldırım at Ege University, Izmir, Turkey. The voucher specimen has been deposited at the herbarium of Ege University with voucher no EGE42372 (for *S. albiflora*) and EGE42377 (for *S. leptoclada*). 

**Table 1:** Chemical composition of the essential oils of *Sideritis albiflora *and *S. leptoclada*

**RI ** **a**	**Compounds**	***S. albiflora *** **(%** **b** **)**	***S. leptoclada ***(**%b**)	**Identification Methods**
914	*α*-Pinene	0.1	0.7	Co-GC, MS, RI
942	1-octen-3-ol	0.6	-	Co-GC, MS, RI
954	3-octanol	0.2	-	Co-GC, MS, RI
960	*β*-Pinene	*tr*	1.3	Co-GC, MS, RI
989	*α*-Phellandrene	0.3	0.1	Co-GC, MS, RI
1000	Benzeneacetaldehyde	0.1	-	MS, RI
1023	*p*-Cymene	*tr*	-	Co-GC, MS, RI
1025	Limonene	0.8	0.4	Co-GC, MS, RI
1026	*β*- Phellandrene	2.0	1.8	Co-GC, MS, RI
1046	*α*-Terpinene	0.2	0.1	Co-GC, MS, RI
1074	Terpinolene	-	0.2	Co-GC, MS, RI
1109	Linalol	-	0.9	Co-GC, MS, RI
1068	Linalool oxyde	0.1	-	Co-GC, MS, RI
1075	2-Nonen-1-ol	0.8	-	Co-GC, MS, RI
1106	*cis-*Sabinol	-	0.2	MS, RI
1131	*cis*-Verbenol	0.2	0.1	MS, RI
1159	Terpinene-4-ol	1.0	0.4	Co-GC, MS, RI
1169	*α*-Terpineol	1.0	0.9	Co-GC, MS, RI
1175	Verbenone	-	0.1	MS, RI
1177	Myrtenol	-	0.3	Co-GC, MS, RI
1168	*trans*-Carveol	-	0.1	MS, RI
1250	Nerol	0.4	0.4	Co-GC, MS, RI
1252	Linalyl acetate	2.2	*tr*	MS, RI
1284	Bornyl acetate	-	0.4	Co-GC, MS, RI
1286	Thymol	1.5	0.2	Co-GC, MS, RI
1295	Carvacrol	6.0	2.4	Co-GC, MS, RI
1338	Eugenol	1.2	0.4	MS, RI
1347	*α*-Cubebene	-	0.2	MS, RI
1360	Domascenone	-	1.2	MS, RI
1371	*α*-Copaene	*tr*	4.6	Co-GC, MS, RI
1381	*β*-Bourbonene	0.6	1.9	Co-GC, MS, RI
1385	*β*-Cubebene	4.3	*tr*	MS, RI
1390	*β*-Elemene	-	1.2	MS, RI
1408	*α*-Gurjunene	0.3	1.5	MS, RI

**Table 1:** Chemical composition of the essential oils of *Sideritis albiflora *and *S. leptoclada*

**RI ** a	**Compounds**	***S. albiflora *** **(%** b **)**	***S. leptoclada ***(**%**^b^)	**Identification Methods**
1448	*α*-Humulene	1.4	*tr*	Co-GC, MS, RI
1452	*β*-Caryophyllene	**21.2**	**17.0**	Co-GC, MS, RI
1456	Alloaromadendrene	1.0	0.6	Co-GC, MS, RI
1474	Germacrene D	*tr*	**17.9**	MS, RI
1487	*τ*-Gurjunene	**13.6**	6.3	MS, RI
1489	*α*-Murolene	-	0.5	MS, RI
1512	Nerolidol	2.5	-	Co-GC, MS, RI
1546	*δ*-Cadinene	0.8	**16.6**	MS, RI
1549	Spathulenol	1.6	-	Co-GC, MS, RI
1561	Caryophyllene oxide	9.0	5.4	Co-GC, MS, RI
1578	Viridiflorol	6.0	-	MS, RI
1590	Ledol	3.4	2.1	MS, RI
1598	Alloaromadendrene oxide	-	0.8	MS, RI
1608	*δ*-Cadinol	-	3.2	MS, RI
1678	2-Tridecenoic acid	-	0.9	MS, RI
1739	Tetradecanoic acid	1.0	1.6	Co-GC, MS, RI
1833	Hexahydrofarnesyl acetone	2.0	2.2	MS, RI
1850	Pentadecanoic acid	**-**	0.1	Co-GC, MS, RI
1895	Farnesyl acetone	0.1	-	MS, RI
1901	2-Heptadecanone	**-**	1.3	MS, RI
1977	Manoyl oxide	**-**	1.2	MS, RI
2001	Palmitic acid	**12.3**	*tr*	Co-GC, MS, RI
2112	Oleic acid	**-**	0.2	Co-GC, MS, RI
2300	Tricosane	*tr*	-	MS, RI
2400	Tetracosane	*tr*	-	MS, RI
	Monoterpene hydrocarbons	3.2	4.6	
	Monoterpenoids	13.7	6.8	
	Sesquiterpene hydrocarbons	43.2	69.5	
	Sesquiterpenoids	22.5	11.5	
	Others	17.1	7.5	
	Total identified (%)	99.7	99.9	
	Total number of compounds	41	47	

**Co-GC** **: **Co-injection with authentic compounds,** RI: **Retention Index literature comparison,** tr**: trace.

a **: **Kovats index on DB–5 fused silica column,

b **: **Percentage concentration,

**Table 2:** Antioxidant activities of the essential oils of *S. albiflora *and *S. leptoclada *by β-carotene-linoleic acid, DPPH•, ABTS•+, CUPRAC and metal chelating assays^a^

Antioxidant Activity						
		β-carotene-linoleic acid assay	DPPH^• ^assay	ABTS^•+^ assay	CUPRAC assay	Metal chelating activity
		IC50 (μg /mL)	Inhibition %^b^	IC50 (μg/mL)	A0.50 (μg/mL)^c^	Inhibition %^b^
***Sideritis *** **species**	***S. albiflora***	**73.8±0.8**	**28.3±0.1**	**50.6±1.0**	**181.7±0.6**	***NA*** f
	***S. leptoclada***	**86.5±0.8**	**17.9±0.9**	**>200**	**>200**	***NA*** f
**Standards**	**α** ***-Tocopherol*** d	**2.1±0.1**	**96.7±0.1**	**4.3±0.1**	**66.7±0.0**	***NT*** e
	***BHA*** d	**1.3±0.0**	**94.1±0.1**	**4.1±0.0**	**24.4±0.7**	***NT*** e
	**EDTA** d	***NT*** e	***NT*** e	***NT*** e	***NT*** e	**96.3±0.1**

a : IC values represent the means ± SEM of three parallel sample measurements (p < 0.05).

b : % inhibition of 800 µg/mL concentration of samples.

c : A values represent the means ± SEM of three parallel sample measurements (p < 0.05).

d :Reference compound.

e :NT: not tested.

f :NA: not active.

**Table 3:** Total phenolic and total flavonoid contents of the essential oils of *S. albiflora *and *S. leptoclada*^a^

***Sideritis species***	**Total phenolic contents (µg PEs/mg DW** b **)**	**Total flavonoid contents (µg QEs/mg DW** c **)**
*S. albiflora*	41.5±0.8	21.4±1.0
*S. leptoclada*	32.5±0.5	5.5±0.3

a Values expressed are means ± S.D. of three parallel measurements. (p < 0.05)

b PEs, pyrocatechol equivalents.

c QEs, quercetin equivalents.

**Table 4:** Anticholinesterase and anti-tyrosinase activities of the essential oils of *S. albiflora and S. leptoclada.*^a^

**Anticholinesterase activity**				**Anti-tyrosinase activity**
	**AChE assay (Inhibition %** b **)**		**BChE assay (IC** **50 ** **(µg/mL))**	**Tyrosinase assay (Inhibition %** b **)**
*Sideritis* species	*S. albiflora*	22.1±0.4	157.2±0.9	15.2±0.4
	*S. leptoclada*	4.3±0.3	199.0±1.0	*NA* e
	Galantamine^c^	80.4±0.4	50.8±0.9	*NT* d
Standards				
	Kojic acid^c^	*NT* d	*NT* d	83.6±0.2

a IC values represent the means ± standard deviation of three parallel measurements (p < 0.05).

b % inhibition of 200 µg/mL concentration of *Sideritis *essential oils.

c Reference compounds.

d NT: not tested.

e NA: not active.

*Spectral measurements and chemicals used*

Bioactivity measurements were carried out on a 96-well microplate reader, SpectraMax 340PC384 (Molecular Devices, Silicon Valley, CA). The measurements and calculations of the activity results were evaluated by using Softmax PRO v5.2 software (Molecular Devices, Silicon Valley, CA). Qualitative and quantitative analysis of the essential oils were performed using GC (Shimadzu GC-17 AAF, V3, 230V series gas chromatography, Japan) and GC/MS (Varian Saturn 2100T, USA).

Pyrocatechol, quercetin, ferrous chloride, copper (II) chloride and ethylenediaminetetraacetic acid (EDTA) were purchased from E. Merck (Darmstadt, Germany). Butylatedhydroxyl anisole (BHA), α-tocopherol, β-carotene, polyoxyethylene sorbitan monopalmitate (Tween-40), linoleic acid, Folin–Ciocalteu’s reagent (FCR), neocuproine, 1,1-diphenyl-2-picryl-hydrazyl (DPPH), 2,2′-azino *bis *(3-ethylbenzothiazoline-6-sulfonic acid) diammonium salt (ABTS), 3-(2-pyridyl)-5,6-di(2-furyl)-1,2,4-triazine-5′,5′′-disulfonic acid disodium salt (Ferene), acetylcholinesterase (AChE) from electric eel (Type-VI-S, EC 3.1.1.7, 425.84 U/mg, Sigma, St. Louis, MO), butyrylcholinesterase (BChE) from horse serum (EC 3.1.1.8, 11.4 U/mg, Sigma, St. Louis, MO), tyrosinase from mushroom (EC 232-653-4, 250 KU, ≥1000 U/mg solid, Sigma), L-DOPA (3,4-Dihydroxy-D-phenylalanine), kojic acid, 5,5′-dithio*bis *(2-nitrobenzoic) acid (DTNB), galantamine, acetylthiocholine iodide, and butyrylthiocholine chloride were purchased from Sigma Chemical Co. (Sigma-Aldrich GmbH, Sternheim, Germany). All other chemicals and solvents were of analytical grade.

*Isolation of the essential oil*

The essential oils of dried aerial parts of *S. albiflora* and *S. leptoclada* were extracted via the hydro-distillation by Clevenger type apparatus for 4 h. The oils were dried over anhydrous sodium sulphate and stored under + 4 °C until analysed.

*Analysis of the essential oil*

*Gas chromatography (GC)*

A Flame Ionization Detector (FID) and a DB-5 fused silica capillary non-polar column (30 m×0.25 id., film thickness 0.25 μm) were used for GC analyses. The injector temperature and detector temperature were adjusted 250 and 270 °C, respectively. Carrier gas was He at a flow rate of 1.4 mL/min. Sample size was 1.0 μL with a split ratio of 20:1. The initial oven temperature was held at 60 °C for 5 min, then increased up to 240 °C with 4 °C/min increments and held at this temperature for 10 min. The percentage composition of the essential oil was determined by the ClassGC10 GC computer program.

*Gas chromatography–mass spectrometry (GC–MS)*

An Ion trap MS spectrometer and a DB-5ms fused silica non-polar capillary column (30 m×0.25 mm ID, film thickness 0.25 μm) were used for the GC–MS analyses. Carrier gas was helium at a flow rate of 1.4 mL/min. The oven temperature was held at 60 °C for 5 min, then increased up to 240 °C with 4 °C/min increments and held at this temperature for 10 min. Injector and MS transfer line temperatures were set at 220 °C and 290 °C, respectively. The ion source temperature was 200 °C. The injection volume was 0.2 μL with a split ratio of 1:20. EI–MS measurements were taken at 70 eV ionization energy. Mass range was from m/z 28 to 650 amu. Scan time 0.5 s with 0.1 inter scan delays. Identification of components of the essential oils was based on GC retention indices and computer matching with the Wiley, NIST-2005 and TRLIB Library as well as by comparison of the fragmentation patterns of the mass spectra with those reported in the literature and whenever possible, by co-injection with authentic compounds (34).

*Total phenolic and flavonoid content*

The phenolic content in essential oils were expressed as microgram of pyrocatechol equivalents (PEs), determined with FCR according to the method of Slinkard and Singleton (35) as described in the literature. The phenolic contents were calculated according to the following equation that was obtained from standard pyrocatechol graph: 

Absorbance=0.002[pyrocatechol (µg)] + 0.0015 (*r*^2^: 0:9999)

Measurement of flavonoid content of the essential oils was based on the aluminium nitrate method and results were expressed as microgram of quercetin equivalents (36). The flavonoid contents were calculated according to following equation that was obtained from the standard quercetin graph:

Absorbance = 0.014[quercetin (µg)] – 0.029 (*r*^2^: 0:9979)

*Antioxidant activity*

*β-carotene/linoleic acid assay*

The total antioxidant activity was evaluated using β-carotene-linoleic acid test system with slight modifications (37,38). The sample concentration providing 50 % lipid peroxidation inhibition activity (IC_50_ μg/mL) was calculated from the graph of antioxidant activity percentages (Inhibition %) against sample concentrations (μg/mL).

*DPPH free radical scavenging assay*

The free radical scavenging activity was determined spectrophotometrically by the DPPH assay described by Blois (39). The sample concentration providing 50 % radical scavenging activity (IC_50_ μg/mL) was calculated from the graph of antioxidant activity percentages (Inhibition %) against sample concentrations (μg/mL).

*ABTS cation radical scavenging Assay*

The spectrophotometric analysis of ABTS^•+ ^scavenging activity was determined according to the method of Re *et al.* (40). The sample concentration providing 50 % radical scavenging activity (IC_50_ μg/mL) was calculated from the graph of antioxidant activity percentages (Inhibition %) against sample concentrations (μg/mL).

*Cupric reducing antioxidant capacity (CUPRAC) assay*

The cupric reducing antioxidant capacity was determined according to the method of Apak *et al.* (41). Results were given as A_0.50 _which corresponds to the concentration providing 0.500 absorbance. The sample concentration providing 0.500 absorbance (A_0.50 _μg/mL) was calculated from the graph of the absorbance signal of cupric reducing antioxidant capacity against the sample concentration (μg/mL). 

*Metal chelating assay*

The chelating activity of the essential oils on Fe^2+^ was spectrophotometrically measured (42). EDTA was used as standard for comparison of the activity. The results were given as inhibition percentage (%) at 200 µg/mL concentration of the essential oils. 

*Enzyme inhibitory activity*

*Cholinesterase inhibition*

Acetylcholinesterase and butyrylcholinesterase inhibitory activity was measured slightly modifying the spectrophotometric method developed by Ellman *et al.* (43). Galantamine was used as reference compound. The percentage inhibition of the enzyme and IC_50 _values of the essential oils were calculated from the graph of anticholinesterase inhibitory activity percentages (Inhibition %) against sample concentrations (μg/mL).

*Tyrosinase inhibition*

Tyrosinase enzyme inhibitory activity was measured by the spectrophotometric method as described by Masuda *et al.* (44). Kojic acid was used as the reference compound. The results were given as inhibition percentage (%) of the enzyme at 200 µg/mL concentration of the essential oils. 

*Statistical analysis*

All data on antioxidant and enzyme inhibitory activity tests were the average of three parallel sample measurements. Data were recorded as mean ± S.E.M. Significant differences between means were determined by student’s-*t* test, *p* values < 0.05 were regarded as significant.

## Results and Discussion

*Chemical composition*

The chemical composition of the essential oils of *S. albiflora* and *S. leptoclada* were analysed by GC and GC-MS. The investigated essential oils obtained from the dried aerial parts of *S. albiflora* and *S. leptoclada* were with yields of 0.06 and 0.03 % (v/w) on dry weight basis, respectively. The chemical composition of the essential oils, relative percentage (%) and Kovats index of compounds are given in **Table 1**. Forty-one compounds were identified in the essential oil of *S. albiflora* representing 99.7% of the total oil; the main compound was *β*-caryophyllene (21.2%), followed by *τ*-gurjunene (13.6%), palmitic acid (12.3%), caryophyllene oxide (9.0%), viridiflorol (6.0%), and carvacrol (6.0%). Forty-seven compounds were identified in essential oil of *S. leptoclada*, which represent 99.9% of the total oil; the major compound was Germacrene D (17.9%), followed by *β*-caryophyllene (17.0%), *δ*-cadinene (16.6%), *τ*-gurjunene (6.3%) and caryophyllene oxide (5.4%).

Sesquiterpene hydrocarbons were the most abundant compounds in both essential oils (43.2 % for *S. albiflora* and 69.5 % for *S. leptoclada*) and followed by sesquiterpenoids (22.5 % for *S. albiflora* and 11.5 % for *S. leptoclada*). 

Topçu *et al.* (21) were previously studied the essential oil composition of *S. albiflora *by using thermal desorption GC-MS and headspace GC-MS techniques. The essential oil was recorded as rich in sesquiterpene and *β-*caryophyllene, *γ*-cadinene, *α*-pinene, pulegone,* β*-pinene and caryophyllene oxide were identified as major compounds. In our study, *α*-pinene and *β*-pinene were found in small quantities, *γ*-cadinene and pulegone were not identified. In recent studies, essential oil composition of *S. brevibracteata* was determined and *β*-caryophyllene, germacrene D and *α*-cadinene were identified as major compounds (45). In other studies, the major compounds of the essential oil of *S. galactica* were found as *α*-pinene, *β-*pinene and *β*-caryophyllene by Zengin *et al.* (46) and *epi*-cubebol, *trans*-piperitol and carvone were found as major compounds in the essential oil of *S. cypria* by Hanoğlu *et al.* (47). When our results were compared to literature findings, the differences and similarities were found in the chemical composition of the essential oil of *S. albiflora and S. leptoclada.* These differences in quantity and quality of the essential oils could be derived from different parts, soil climatic factors, and methods (48). In this study, the chemical composition of essential oil of *S. leptoclada* was studied in great detail for the first time.

*Antioxidant activity*

*Sideritis* essential oils were tested for their antioxidant activities employing five different *in-vitro *assay systems i.e. β-carotene-linoleic acid, DPPH radical scavenging, ABTS cation radical scavenging, cupric-reducing antioxidant capacity (CUPRAC) and metal chelating activity. Table 2 shows the antioxidant activities of the essential oils of *Sideritis* species compared with BHA, α-tocopherol and EDTA. The essential oils were tested at different concentrations and IC_50 _values were determined.

Total antioxidant capacities of the essential oils of *Sideritis *species were tested by the β-carotene-linoleic acid assay. This is one of the rapid methods to screen antioxidants and the lipid peroxidation inhibitors. This method is mainly based on the principle that linoleic acid gets oxidized by reactive oxygen species produced by oxygenated water. β-carotene oxidation is activated by the formed products will lead to discoloration. Antioxidants cause a decrease in the discoloration and the decrease is measured at 470 nm spectrophotometrically (49). The essential oil of *S. albiflora *exhibited the highest lipid peroxidation inhibitory activity with IC_50 _values of 73.8 ± 0.8 µg/mL (Table 2).

The radical scavenging activities of *Sideritis* essential oils were determined by DPPH^•^ and ABTS^•+^ radicals. The results of DPPH^•^ and ABTS^•+^ assays were also given in Table 2. In DPPH^• ^and ABTS^•+^ assays, the essential oil of *S. albiflora* showed the higher radical scavenging activity with inhibition value of 28.3 ± 0.1 % and IC_50 _value of 50.6 ± 1.0 µg/mL, respectively (Table 2). 

The reducing power of the *Sideritis* essential oils was determined by CUPRAC assay. CUPRAC assay is an easy method to completely define the reducing capacity of the antioxidants. In this method, the absorbance is measured at 450 nm by the occurrence of a stable complex between copper (I) ions and neocuproine, the latter is created by the reduction of copper (II) in the existence of neocuproine (50). In other words, the highest absorbance shows the highest activity. Similar to radical scavenging and lipid peroxidation inhibitory activities, the essential oil of *S. albiflora* (A_0.05_: 181.7 ± 0.6 µg/mL) was found to be higher reductant than the essential oil of *S. leptoclada *(A_0.05_ > 200 µg/mL) (Table 2). 

Metal chelating activity is important to test to a pro-oxidant effect of transition metals on the lipid oxidation. For instance, the ferrous state of iron accelerates lipid oxidation by breaking down hydrogen and lipid peroxides to reactive free radicals through the Fenton Reaction (51). However, none of the essential oils showed any chelating activity (Table 2).

In the recent studies antioxidant activities of the essential oils of *Sideritis* species were investigated. Venditti *et al.* (52) studied antioxidant activity of the essential oil of *S. montana* by using DPPH^• ^and ABTS^•+^ assays. Essential oil was reported to have low activity in ABTS^•+^ assay and no activity in DPPH^• ^assay. In another study, antioxidant capacity of the essential oil of* S. galactica *was tested by free radical scavenging (DPPH, ABTS and NO), reducing power (FRAP and CUPRAC), metal chelating and phosphomolybdenum methods and the essential oil exhibited moderate activity in all studied methods (46). The obtained activity results are in accordance with the literature.

*Total phenolic and total flavonoid content*

The total phenolic and flavonoid contents of the essential oils of *Sideritis* species were determined as pyrocatechol and quercetin equivalents, respectively. The essential oil of *S. albiflora* has the higher level of the phenolic compounds (41.5 ± 0.8 µg PEs/mg) and flavonoid compounds (21.4 ± 1.0 µg QEs/mg). In addition, the essential oil of *S. albiflora* with higher concentrations of phenolic and flavonoid contents showed the highest activity in all antioxidant activity assays (Table 3).

*Cholinesterase enzyme inhibition*

Various pathogenic factors such as aggregated amyloid-β-peptide and tau protein, oxidative stress, excessive transition metals, and reduced acetylcholine levels cause Alzheimer’s disease (53). Acetylcholinesterase ends the effect of this neurotransmitter at cholinergic synapses by hydrolyzing acetylcholine to choline and acetate. For this reason, in the patients, AChE is the target of cholinesterase inhibitors used for the treatment. In the method, the enzyme hydrolyses the substrate acetylthiocholine to thiocholine which reacts with DTNB to produce 2-nitrobenzoate-5-mercaptothioclonine and 5-thio-2-nitrobenzoate which is detected at 412 nm. Table 4 shows inhibition % of 200 µg/mL concentration of *Sideritis *essential oils for AChE assay and IC_50 _values of *Sideritis* essential oils for BChE assay. The essential oil of *S. albiflora* (22.1 ± 0.4 %) exhibited five times higher acetylcholinesterase activity than the essential oil of *S. leptoclada* (4.3 ± 0.3 %). Against BChE enzyme, the essential oils of *S. albiflora *and *S. leptoclada *showed moderate inhibitory activity with IC_50 _values of 157.2 ± 0.9 µg/mL and 199.0 ± 1.0 µg/mL, respectively. In the literature survey, no study was found related to cholinesterase inhibition activity of *Sideritis* essential oils. 

*Tyrosinase enzyme inhibition*

Tyrosinase is a cupper-containing enzyme mostly found in animals and plants. Also, it is responsible for enzymatic browning reactions in damaged fruits. Therefore, investigations about discovering new tyrosinase inhibitors are increasing for using in foods and cosmetics (54). This method is based on the measurement of the absorbance of dopachrome as a result of the reaction between L-tyrosine and L-DOPA. Results were given as inhibition (%) at 200 µg/mL concentration. As can be seen from Table 4, *S. albiflora* (15.2 ± 0.4 %) essential oil showed mild anti-tyrosinase activity while *S. leptoclada *essential oil did not exhibit any activity. To our knowledge, there is no study regarding anti-tyrosinase activity of *Sideritis *essential oils in the literature.

## Conclusion

The antioxidant, anticholinesterase and anti-tyrosinase activities together with total phenolic and total flavonoid contents of the essential oils of *Sideritis albiflora* and *S. leptoclada* were reported here for the first time. The essential oil of *S. albiflora* including high concentrations of phenolic and flavonoid contents indicated the highest antioxidant and enzyme inhibitory activities. 

The consumption of *Sideritis* species may protect individuals without side effects against melanogenesis, amnesia and oxidative stress. However, further studies, particularly *in vivo* activity tests on essential oils and explored to new drug candidates from these species are still needed.
